# Cardiac Autonomic Imbalance in Newly Diagnosed and Established Diabetes Is Associated with Markers of Adipose Tissue Inflammation

**DOI:** 10.1155/2012/878760

**Published:** 2011-11-01

**Authors:** David C. Lieb, Henri K. Parson, Gregg Mamikunian, Aaron I. Vinik

**Affiliations:** ^1^Division of Endocrinology and Metabolism, Department of Medicine, Eastern Virginia Medical School, Strelitz Diabetes Center for Endocrine and Metabolic Disorders, 855 West Brambleton Avenue, Norfolk, VA 23510, USA; ^2^Inter Science Institute, 944 West Hyde Park Boulevard, Inglewood, CA 90302, USA

## Abstract

*Introduction*. Diabetics die from cardiovascular disease at a much greater rate than nondiabetics. Cardiac autonomic imbalance predicts increased cardiovascular risk and mortality. We studied the relationship between cardiac autonomic imbalance and adipose tissue-derived inflammation in newly diagnosed and established type 2 diabetes. *Materials and Methods*. Non-diabetics, newly diagnosed diabetics, and established diabetics were included. Anthropomorphic and biochemical measurements were obtained, and insulin resistance was approximated. Cardiac autonomic function was assessed using conventional measures and with power spectral analysis of heart rate. *Results and Discussion*. Heart rate variability was reduced in all diabetics. Interleukin-6 was higher in diabetics, as was the high molecular weight adiponectin-to-leptin ratio. Interleukin-6 correlated negatively with measures of autonomic balance. Ratios of adiponectin to leptin correlated positively with measures of autonomic balance. Cardiac autonomic imbalance and inflammation occur early in diabetes and are interrelated. *Conclusions*. Cardiac autonomic imbalance correlates with the adipose tissue-derived inflammation seen early in type 2 diabetes.

## 1. Introduction

Greater than 50% of adults with type 2 diabetes (T2D) have coronary artery disease (CAD), and death from CAD is two- to five-times more likely in a diabetic than in a nondiabetic patient [1]. Many individuals with diabetes have silent myocardial ischemia, and may succumb to cardiovascular disease (CVD) before presenting for further evaluation and treatment. As demonstrated by the Detection of Silent Myocardial Ischemia in Asymptomatic Diabetic Subjects (DIAD) study, traditional risk factors for CVD such as dyslipidemia and hypertension do not accurately predict all individuals with silent myocardial ischemia [2]. Forty percent of patients with silent ischemia are missed using only these traditional risk factors. Clearly, better predictors of myocardial ischemia are needed. 

 Cardiovascular autonomic neuropathy (CAN), defined as abnormalities of the cardiovascular system as a result of damage to the autonomic fibers innervating the heart and vasculature, is associated with increased major cardiovascular events, as well as increased mortality after a myocardial infarction, in diabetic patients [3–5]. In the DIAD study, a diminished Valsalva heart rate ratio (a measure of CAN) was strongly associated with silent myocardial ischemia, independent of more traditional risk factors including gender, age, hypertension, and smoking [2]. Symptoms of more generalized autonomic neuropathy were also associated with myocardial ischemia, including orthostatic hypotension, erectile dysfunction, and postmeal bloating. In the European Epidemiology and Prevention of Diabetes (EURODIAB) study, autonomic dysfunction was present in one-third of type 1 diabetic (T1D) subjects, and was strongly associated with coexisting CVD after adjustment for age, hemoglobin A1c (HbA1c), and duration of diabetes [6]. Results from the recently reported Action to Control Cardiovascular Risk in Diabetes (ACCORD) trial, which showed an increased mortality in T2Ds at increased risk for CVD randomized to receive intensive glycemic control, demonstrated that individuals with CAN were 1.55- to 2.14-times as likely to die (all-cause mortality) during the study when compared to those without CAN [7]. Death from CVD (including that related to arrhythmia, myocardial infarction, heart failure, cardiovascular interventions, and stroke) was even more likely (1.94–2.95-times) in subjects with CAN. Together, these data suggest that factors indicating autonomic dysfunction may be better predictors of cardiac events than more traditional risk factors, and their early identification could prove to be salutary for CVD prevention [8].

 Inflammation plays a key role in the development of both T2D and atherosclerosis. Obesity, a well-known risk factor for both diabetes and CVD, is an inflammatory condition, and adipose tissue, especially abdominal or visceral fat, produces a variety of proinflammatory cytokines involved in the development of insulin resistance and atherosclerosis [9]. These proinflammatory “adipokines,” including interleukin-6 (IL-6) and tumor necrosis factor-alpha (TNF-*α*), lead to increased release of free fatty acids (FFA) from adipose tissue that stimulate increased hepatic production of proatherogenic lipids such as very-low-density lipoprotein (VLDL), and prothrombotic proteins such as plasminogen activator inhibitor-1 (PAI-1) [10]. The adipocyte-derived hormones leptin and adiponectin are also involved in energy balance, and adiponectin in particular may have anti-inflammatory properties that are lost or reduced in the obese, diabetic patient [11]. 

 The relationship between autonomic balance and adipose tissue-derived inflammation in T2D is not entirely clear, though both play important roles in the CVD that affects a majority of individuals with this disease, and cardiac autonomic imbalance predicts cardiovascular risk in diabetic patients [12]. In our case-control study involving nondiabetic, newly diagnosed T2D, and established T2D obese subjects, we measured cardiac autonomic function and circulating adipose tissue-derived inflammatory cytokines to determine if there was indeed such a relationship. We hypothesized that there would be significant differences between our groups in regard to both autonomic function and markers of inflammation, and that autonomic imbalance would be associated with “sick,” or inflammatory adipose tissue. We report here a number of exciting differences between people with newly diagnosed diabetes, established diabetes, and healthy controls and significant correlations between inflammation and autonomic balance in early and established T2D that have implications for both the determination of CVD risk in T2D patients, and for the treatment and reduction of that risk.

## 2. Materials and Methods

### 2.1. Study Participants

 Subjects were recruited from a population in Norfolk, Virginia through the Strelitz Diabetes Center at Eastern Virginia Medical School. Three groups were recruited: healthy controls with no prior history of diabetes, individuals with newly diagnosed diabetes (duration less than 6 months), and those with established diabetes (duration greater than 6 months, with a mean duration of 11 years). Diabetes was defined using current American Diabetes Association criteria and/or the need for anti-diabetic medications to control blood glucose. Exclusion criteria included the presence of type 1 diabetes, diabetic retinopathy, diabetic kidney disease, untreated hypothyroidism, liver disease, congestive heart failure, a history of major macrovascular events (including stroke and myocardial infarction within the previous 3 months), significant nondiabetic neuropathy, and/or lower extremity ulcerations or amputations. Anthropomorphic measurements were obtained for each subject, including body mass index (BMI), percent (%) body fat, waist circumference and hip circumference (for calculation of waist-to-hip ratio), systolic and diastolic blood pressure, and heart rate (HR). 

### 2.2. Laboratory Assessment and Homeostasis Model 2 Assessment (HOMA2) Calculations

 Each subject had a fasting laboratory evaluation, including serum glucose, triglycerides, total cholesterol, high-density and low-density lipoprotein cholesterol, C-peptide, HbA1c, and insulin. Markers of inflammation including IL-6, TNF-*α*, and PAI-1 were measured, as were the adipokines leptin and adiponectin (both total (TA) and high-molecular weight forms (HMW)). Analyte specific reagents (ASR) were obtained for IL-6, TNF-*α*, PAI-I, leptin, and adiponectin (total and high molecular weight). The specific analytes were validated at Inter Science Institute Laboratory, in accordance with standard protocol for validations. EIA, Elisa, and RIA kits that were commercially available for these analytes presented a more challenging task for validation than the ASR approach. Adiponectin-to-leptin ratios were then calculated [13]. Determinations of beta cell function and insulin resistance were made using the homeostasis model assessment 2 (HOMA2) [14] (calculator available from the University of Oxford Diabetes Trials Unit; http://www.dtu.ox.ac.uk/homacalculator/index.php). The study protocol was approved by the institutional review board at Eastern Virginia Medical School.

### 2.3. Autonomic Nervous System Function Assessment

 Autonomic nervous system (ANS) function can be assessed by the measurement of heart rate variability (HRV), which is reduced early in the development of CAN in diabetic patients, and has been associated with increased risk for death after myocardial infarction [3, 15, 16]. Time- and frequency-domain analyses are techniques for assessing HRV that are used in both the research and clinical settings. Time-domain analysis of HRV provides a measure of the sympathetic and parasympathetic control of the heart beat (the R-R interval on an electrocardiogram) recorded with maneuvers including deep breathing, the Valsalva maneuver, and standing from the supine position while frequency-domain analysis is performed under resting conditions [3, 15]. Given their complementary nature both time- and frequency-domain analyses were utilized in this study.

 Subjects were instructed to sit with their feet flat on the floor, and a blood pressure cuff was placed on the upper left arm and electrodes were placed on the chest. Subjects were instructed not to speak during the examination, and were instructed in how to perform a Valsalva maneuver prior to starting. Subjects were told to breathe normally (relaxed breathing) for 5 minutes, followed by 1 minute of slow, deep breaths (inspiration and expiration for approximately 5 seconds each). Subjects took regular breaths for 1 minute, and then performed 5 consecutive Valsalva maneuvers (each lasting approximately 10 seconds), followed by 2 minutes of regular breathing. Finally, the subjects were asked to stand while taking regular breaths. Heart rate and blood pressure were monitored during the examination. Measures of autonomic function, including responses in HRV to deep breathing (expiratory/inspiratory ratio; E/I), Valsalva maneuver (Valsalva ratio), and standing (postural ratio), were recorded. These ratios have been described in detail [15, 17, 18].

 Power spectral analysis of HRV was performed under resting conditions (ANSAR; ANX 3.0 software; ANSAR Group, Inc., Philadelphia, PA) with demonstration of low-frequency (LF) and high-frequency (HF) components. Data using ANSAR were normalized. The LF component of the power spectrum of HRV primarily reflects sympathetic activity while the HF component (also termed the respiratory-frequency, RF) primarily reflects parasympathetic activity [3]. LF/RF ratios were calculated providing a measure of sympathetic/parasympathetic activity. The total spectral power (TSP) was calculated, as was the standard deviation of all normal R-R intervals (sdNN), a measure of both sympathetic and parasympathetic action on HRV, and the root-mean square of the difference of successive R-R intervals (rmSSD), a measure primarily of parasympathetic activity [15]. 

### 2.4. Statistical Analysis

 All data are presented as mean ± SEM. Analysis of variance (ANOVA) was used to compare differences in baseline characteristics, autonomic function, adipokines, *β*-cell function, and insulin sensitivity results between groups. If significance was observed, a Tukey-Kramer post hoc analysis was performed to determine which groups differed. To determine correlations between autonomic function and adipokines, a Spearman's rank correlation test was used. In cases where the data were not normally distributed, the Wilcoxon signed-rank test was used to determine differences between study groups. Comparison of ANS assessment variables was performed after age adjustment since age has been shown to affect HRV [19]. JMP 8.0 software (SAS Institute, Inc., Cary, North Carolina) was used for all statistical analyses. Significance was accepted at the *P* < 0.05 level for all statistical analyses. 

## 3. Results and Discussion

### 3.1. Baseline Characteristics

 Baseline characteristics are shown in Table 1. Fifteen subjects were recruited to each group (nondiabetic controls, newly diagnosed diabetics, and established diabetics). As expected the diabetic subjects had higher HbA1c values (established 7.9 ± 0.3%; newly diagnosed 7 ± 0.4%; controls 5.3 ± 0.2%; *P* < 0.0001). The diabetic subjects had a greater percent body fat when compared with those in the control group (established 38.7 ± 2.1%; newly diagnosed 37.1 ± 2.0%; controls 26.4 ± 2.9%; *P* < 0.01). LDL cholesterol was significantly higher in the control subjects (control 3.34 ± 0.17; newly diagnosed 2.59 ± 0.31; established 2.39 ± 0.21 mmol/L; *P* < 0.05).

### 3.2. Measures of Autonomic Nervous System Function

 Measures of ANS function in each of the three groups are shown in Table 2. The newly diagnosed and established diabetics had reduced HRV with deep breathing when compared with the controls (1.20 ± 0.03 and 1.17 ± 0.05 versus 1.26 ± 0.04, *P* < 0.05). The established diabetic subjects had a significantly lower Valsalva ratio when compared with the newly diagnosed and control subjects (1.24 ± 0.05 versus 1.37 ± 0.06 and 1.58 ± 0.21, *P* < 0.05). 

Spectral analysis of the HRV in the three groups revealed several significant differences. Total spectral power (TSP) was significantly lower (indicative of reduced HRV) in the established diabetic subjects (416.95 ± 142.61 versus 950.88 ± 191.95 for controls and 772.94 ± 290.95 for newly diagnosed T2D; *P* < 0.01). Baseline sdNN was also reduced in the established diabetic subjects (28.92 ± 4.65 versus 47.66 ± 4.87 for controls and 41.04 ± 3.92 for newly diagnosed T2D; *P* < 0.001). Baseline rmSSD was significantly lower in the newly diagnosed and established T2D compared with the control subjects (28.77 ± 6.97 for the newly diagnosed T2D and 18.97 ± 3.38 for the established T2D versus 30.18 ± 3.76 for the controls; *P* < 0.05). 

 Together, these data validate previous studies showing reductions in HRV in established diabetes [20]. They also suggest specific measurements of HRV (R-R ratio with deep breathing and rmSSD) that demonstrate abnormalities in autonomic function within 6 months of diabetes diagnosis, and presumably, earlier in the course of the disease. 

### 3.3. Adipose Tissue-Derived Cytokines and Adipokines

 Concentrations for various adipokines are given in Table 3. IL-6 concentrations were significantly higher in the diabetic subjects (newly diagnosed and established) compared with the control, nondiabetic subjects (11.6 ± 2.8 pg/mL for newly diagnosed T2D and 12.0 ± 1.2 pg/mL for established T2D versus 2.8 ± 0.7 pg/mL for controls, *P* < 0.0001). PAI-1 concentrations were significantly higher in the established diabetics compared with the newly diagnosed and control subjects (6.41 ± 1.36 ng/mL for established T2D versus 5.23 ± 0.76 ng/mL for newly diagnosed T2D and 3.05 ± 0.56 ng/mL for controls, *P* < 0.05). 

When analyzed using the Wilcoxon signed-rank test, the total adiponectin-to-leptin ratio (TA/L) was significantly higher in the established T2D when compared with the newly diagnosed diabetics and the controls (0.53 ± 0.32 for the established T2D versus 0.24 ± 0.05 for the newly diagnosed T2D and 1.17 ± 0.76 for the controls, *P* < 0.05). The high-molecular weight adiponectin-to-leptin ratio (HMWA/L) was significantly higher in the established and newly diagnosed diabetics compared with the control subjects (0.07 ± 0.02 for newly diagnosed T2D and 0.26 ± 0.22 for established T2D versus 0.49 ± 0.40 for controls, *P* < 0.05). 

### 3.4. Measures of Insulin Sensitivity/Resistance and Pancreatic Beta Cell Function

 HOMA IR values were not significantly different between the groups and are provided in Table 4. Using HOMA 2%B as a marker, *β*-cell function was significantly increased in the newly diagnosed T2D when compared with the established diabetics and control subjects and significantly impaired in established type 2 diabetes (118.02 ± 13.30 versus 68.31 ± 11.00 for established T2D and 105.16 ± 10.63 for controls, *P* < 0.05). Similar differences were seen when HOMA 2%B was assessed using fasting C-peptide concentrations in place of fasting insulin concentrations (120.74 ± 10.31 versus 85.15 ± 12.57 for established T2D and 111.42 ± 6.85 for controls, *P* < 0.05).

### 3.5. Correlations between Autonomic Function and Inflammation

We found a number of very interesting correlations between measures of autonomic function and adipose tissue-derived inflammatory products in our study (see Table 5). IL-6 correlated negatively with sdNN at baseline. This suggests that elevated levels of this adipocytokine are associated with reductions in HRV seen in patients with diabetes. Of note, IL-6 was significantly elevated in newly diagnosed as well as established diabetics in our study, suggesting that this cytokine may play a role in the autonomic dysfunction that is seen early in the course of diabetes. HMW adiponectin correlated negatively with the LFA/RFA ratio, and an elevated TA/L ratio (and also an elevated HMWA/L ratio) correlated positively with total spectral power and rmSSD. These latter findings demonstrate that the balance of two significant adipokines involved in insulin sensitivity (adiponectin and leptin) may be related to autonomic function.

## 4. Discussion

 This study reveals a number of novel relationships in regard to the autonomic nervous system, adipose tissue-derived inflammation, and the onset and progression of diabetes. We demonstrate ANS dysfunction in newly diagnosed diabetic subjects, as measured by a reduction in R-R variability with deep breathing, as well as by a reduction in rmSSD as measured by HRV through time-domain analysis. Established diabetics also had a reduction in their R-R variability during the Valsalva maneuver, as well as reductions in total spectral power, sdNN, and rmSSD. Newly diagnosed diabetics had higher concentrations of the inflammatory adipokine IL-6, and had low HMW adiponectin-to-leptin ratios compared with control subjects. Established diabetics also had significantly higher concentrations of PAI-1. We found significant correlations between an inflammatory adipokine (IL-6) and measures of autonomic function in our established and newly diagnosed diabetics (sdNN at baseline). We also noted correlations between the HMWA/L ratio and various measures of autonomic function. Our findings suggest that newly diagnosed diabetics have measurable abnormalities in their ANS, and that these changes may be in part regulated through the adipokines IL-6, leptin, and adiponectin but cannot rule out that the effects could be primarily due to autonomic dysfunction with its consequences upon adipose tissue cytokine release. 

 Others have reported abnormal autonomic function in newly diagnosed diabetics. Thirty years ago Fraser et al. reported abnormal ANS function in a group of 10 newly diagnosed diabetics (six of whom were being treated with insulin) [21]. Pfeifer et al. reported abnormal autonomic responses in a group of 33 newly diagnosed diabetic subjects (duration of diabetes less than 12 months), and suggested that abnormal carbohydrate metabolism was a cause of their ANS abnormalities [22]. McDaid et al. described abnormal vasoconstrictor responses to temperature changes and deep-breathing maneuvers in newly diagnosed T2Ds when compared with control subjects [23]. 

 The etiology of ANS dysfunction seen early after the diagnosis of diabetes is not well understood. Some have suggested elevated concentrations of nitric oxide as well as chronic hyperglycemia as causative factors [24, 25]. A recent study from Chang et al. demonstrated that cardiac autonomic dysfunction (as measured by spectral analysis and expiratory/inspiratory ratio) may occur prior to the development of insulin resistance in individuals with 1 or 2 components of the metabolic syndrome [26]. Thus, it is not surprising to find abnormalities in autonomic function, but we add the dimension of change in autonomic balance without necessarily autonomic neuropathy suggesting a potential reversibility at the stage of newly diagnosed diabetes. 

 It has been suggested that adipose tissue becomes “sick” (adiposopathy) in states of obesity, leading to various components of the metabolic syndrome [27]. In our study we build upon this hypothesis, and suggest that adipokines play a role in the development of ANS dysfunction in obese subjects with type 2 diabetes. We demonstrated increased concentrations of the inflammatory protein IL-6 in both newly diagnosed and established diabetic subjects. IL-6 correlated negatively with sdNN, suggesting an association between IL-6 and abnormalities in both the sympathetic and parasympathetic components of the reduced HRV seen in patients with diabetes. März et al. demonstrated that rat sympathetic neurons were able to produce and respond to IL-6, and suggested a possible paracrine role for the interleukin in regulating the sympathetic nervous system (SNS) [28]. Sloan et al. demonstrated a strong inverse relationship between HRV and serum concentrations of IL-6 and CRP in young men and women in the CARDIA study, which looked at risk factors for the development of atherosclerosis [29]. In a group of 264 middle-aged men, Lampert et al. found that IL-6 was inversely associated with various measures of HRV. IL-6 was also associated with multiple cardiovascular risk factors (including BMI, hypertension, and smoking) [30]. The authors suggested that autonomic dysfunction might lead to inflammation, which may then play a role in the development of CAD. Further studies should explore this relationship, and the regulation of IL-6 by both the parasympathetic and sympathetic nervous systems is amenable to examination by determining the impact of adrenergic and cholinergic blockade on IL-6 concentrations. Interestingly, we did not see a significant difference in C-reactive protein (CRP) concentrations between our three groups. CRP has been shown to predict an increased risk for CVD, as well as stroke and noninsulin-dependent diabetes [31]. As CRP production is primarily regulated by IL-6, much of which comes from adipose tissue, it may be that an elevated IL-6 concentration is an earlier predictor of CVD risk than CRP [32]. Our data suggest that measures of cardiac autonomic imbalance, which correlate with IL-6 concentrations, are also early predictors of CVD risk, and may be clinically relevant before more well-known predictors of risk including CRP.

 We found that newly diagnosed diabetic subjects had significantly lower HMW adiponectin/leptin ratios when compared with control subjects, and saw significant correlations between the HMWA/leptin ratio and multiple measures of autonomic function (LFA/RFA ratio, TSP baseline, and rmSSD baseline). Intravenous leptin infusion has been shown to stimulate SNS activity in animal models [33]. In a study of 120 nonobese humans without diabetes, Paolisso et al. demonstrated that increasing plasma leptin concentrations were associated with increasing LFa/HFa ratios, suggesting greater SNS activity as compared with PS activity [34]. Studies have also shown interesting correlations between the adiponectin and autonomic function. In mice, Imai et al. demonstrated that activation of the SNS (via cold temperatures) suppressed the production of adiponectin from white adipose tissue [35]. In humans, Wakabayashi and Aso studied 105 men and women with T2D and found that adiponectin concentrations correlated negatively with the LFa/RFa ratio [36]. Of note, changes in the adiponectin/leptin balance in the course of diabetes may first be seen by utilizing measures of high-molecular weight adiponectin rather than total adiponectin, as demonstrated by our findings (Table 3). These findings and the results in animal studies again suggest that the effects on adipose tissue cytokine release may be a consequence of autonomic dysfunction as opposed to the corollary. 

 Tracey and others have described a neural reflex arc whose afferent arm responds to markers of inflammation, such as cytokines, by eliciting a cholinergic anti-inflammatory effector response, mediated through the parasympathetic nervous system [37]. In animal models of inflammation, inhibition of the afferent arm of this reflex arc (e.g., by vagotomy) leads to increased inflammation and disease severity [38]. Activation of the efferent arm of the reflex arc (through the administration of an acetylcholine receptor agonist) causes a decrease in proinflammatory cytokine production, and a reduction in disease severity [39, 40]. In his review Tracey points out a number of important clinical studies that show correlations between vagal nerve activity and inflammatory human diseases such as rheumatoid arthritis and lupus [37, 41, 42]. Our results are in keeping with this research, and suggest that such a reflex arc may be involved in the inflammation seen early in T2D.

 There are limitations to our study. This is a case-control study with small numbers of subjects. This limits the power of the findings, especially considering that HRV can vary significantly between individuals. However, the differences and correlations demonstrated by the data are highly significant. Also, there were not specific restrictions on whether patients could take medications that might affect ANS function (such as beta-blockers) or anti-inflammatories and future studies would include these exclusionary criteria. Previous studies have demonstrated significant differences in cholesterol concentrations and HOMA IR values between diabetics and nondiabetics [43]. The diabetic subjects in our study had lower cholesterol concentrations than the nondiabetic subjects. We suspect that many of these subjects were taking cholesterol-lowering agents such as statins. Surprisingly HOMA IR did not differ significantly between the diabetic and nondiabetic subjects in our study. This may be a reflection of our small sample size, as the HOMA IR values do differ between the groups, but not significantly. Both adiponectin and leptin increased with progression of diabetes in this study. This might be a result of the increased percent of body fat that these individuals had when compared with the control group. The established diabetic subjects had been diagnosed as having diabetes for an average of 11 ± 1.5 years. It will be important to evaluate subjects with varying durations of diabetes in the future, including a group of prediabetic individuals with impaired glucose tolerance and/or impaired fasting glucose, to determine if they fit with our hypothesis that early hyperglycemia is associated with autonomic dysfunction and changes in adipokine secretion that promote an inflammatory milieu. 

**Figure 1:**
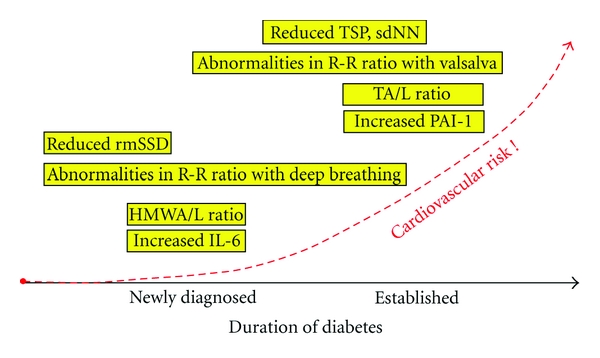
Cardiac autonomic imbalance and inflammation in the progression of diabetes.

 Our study suggests that autonomic dysfunction is seen early in the course of diabetes, and that this occurs alongside alterations in various adipokines, including adiponectin and leptin, and various inflammatory adipocytokines, including IL-6. Future studies could evaluate differences in various inflammatory markers in both subcutaneous and visceral fat in nondiabetic, diabetic, and newly diagnosed diabetic subjects, and seek correlations with autonomic balance and risk factors for CVD in these patients. Future therapies might be directed at reducing the early sympathetic activation seen in newly diagnosed diabetics, with a goal of reducing the impact of the inflammatory response upon the ANS before it leads to the development of CVD thereby reducing CVD and mortality (Figure 1).

## 5. Conclusions

 Here we show that there is strong evidence of inflammation with activation of inflammatory cytokines such as IL-6 and leptin in newly diagnosed type 2 diabetes. These changes correlate with abnormalities in sympathetic-vagal balance. Autonomic dysfunction has been shown to be a predictor of cardiovascular risk and sudden death in patients with type 2 diabetes. A better understanding of the autonomic dysfunction and adipose tissue inflammation seen early in the development of diabetes will lead to further measures for determining which individuals are at the highest risk for cardiovascular disease and mortality, and will also lead to the development of new therapies for reducing that risk.

## Figures and Tables

**Table 1:** Baseline characteristics of study subjects.

Characteristics	Controls	Newly diagnosed T2D	Established T2D	*P* value
	(*n* = 15)	(*n* = 15)	(*n* = 15)	
Age (years)	51.47 ± 2.63	53.53 ± 3.80	55.87 ± 1.99	NS
Diabetes duration (months)	NA	2.80 ± 0.45	132.0 ± 18.55	<0.0001*
Systolic blood pressure (mmHg)	126.73 ± 3.13	129.40 ± 4.9	131.93 ± 3.32	NS
Diastolic blood pressure (mmHg)	77.53 ± 2.01	77.73 ± 2.23	77.6 ± 1.81	NS
Resting heart rate (bpm)	73.67 ± 2.97	66.60 ± 2.74	76.6 ± 3.44	NS
BMI (kg/m^2^)	30.16 ± 2.09	35.27 ± 2.22	35.39 ± 1.68	NS
Waist circumference (cm)	39.10 ± 2.08	44.53 ± 1.87	43.43 ± 1.63	NS
Waist-hip ratio	0.91 ± 0.03	0.95 ± 0.02	0.92 ± 0.02	NS
Percent body fat	26.39 ± 2.91	37.05 ± 2.03	38.71 ± 2.07	0.0012^#^
Fasting plasma glucose (mmol/L)	5.32 ± 0.13	6.12 ± 0.34	7.87 ± 0.84	0.0045*
Fasting plasma insulin (pmol/L)	74.93 ± 11.1	133.3 ± 27.48	115.47 ± 31.06	NS
Fasting C-peptide (nmol/L)	0.70 ± 0.07	1.14 ± 0.15	1.03 ± 0.17	NS
A1C (%)	5.29 ± 0.19	6.95 ± 0.42	7.93 ± 0.33	<0.0001^#^
Fasting total cholesterol (mmol/L)	5.15 ± 0.20	4.87 ± 0.31	4.37 ± 0.24	NS
Fasting triglycerides (mmol/L)	1.02 ± 0.15	1.13 ± 0.17	1.39 ± 0.24	NS
HDL cholesterol (mmol/L)	1.33 ± 0.08	1.41 ± 0.09	1.34 ± 0.13	NS
LDL cholesterol (mmol/L)	3.34 ± 0.17	2.59 ± 0.31	2.39 ± 0.21	0.0157^#^
C-reactive protein (nmol/L)	45.33 ± 18.29	13.91 ± 4.00	43.72 ± 20.76	NS

Data are presented as means ± SE. NA: not applicable; NS: not significant. *Established T2D versus controls/newly diagnosed T2D. ^#^Newly diagnosed/established T2D versus controls.

**Table 2:** Measures of autonomic function.

Measure	Controls	Newly diagnosed T2D	Established T2D	*P* value
	(*n* = 15)	(*n* = 15)	(*n* = 15)	
R-R ratio (deep breathing)	1.26 ± 0.04	1.20 ± 0.03	1.17 ± 0.05	0.0325^#^
R-R ratio (Valsalva)	1.58 ± 0.21	1.37 ± 0.06	1.24 ± 0.05	0.0270*
R-R ratio (standing)	1.29 ± 0.04	1.26 ± 0.05	1.18 ± 0.02	NS
LFa	1.60 ± 0.43	1.90 ± 0.39	1.37 ± 0.37	NS
RFa	1.21 ± 0.27	1.89 ± 0.79	0.73 ± 0.29	NS
LFa/RFa ratio	1.53 ± 0.24	2.51 ± 0.51	2.88 ± 0.56	NS
TSP baseline	950.88 ± 191.95	772.94 ± 290.95	416.95 ± 142.61	0.0023*
sdNN baseline	47.66 ± 4.87	41.04 ± 3.92	28.92 ± 4.65	0.0008*
rmSSD baseline	30.18 ± 3.76	28.77 ± 6.97	18.97 ± 3.38	0.0356^#^

Data are presented as means ± SE. NS: not significant. *Established T2D versus controls/newly diagnosed T2D. ^#^Newly diagnosed/established T2D versus controls. TSP: total spectral power; SDNN: standard deviation of all normal R-R intervals; rmSSD: root mean square of the difference of successive R-R intervals.

**Table 3:** Adipokines in study subjects.

Measurement	Controls	Newly diagnosed T2D	Established T2D	*P* value
	(*n* = 15)	(*n* = 15)	(*n* = 15)	
IL-6 (pg/mL)	2.84 ± 0.68	11.58 ± 2.83	12.04 ± 1.20	<0.0001^#^
	(*n* = 14)		(*n* = 12)	
TNF-*α* (pg/mL)	9.32 ± 2.18	9.14 ± 1.15	27.93 ± 15.4	NS
	(*n* = 14)		(*n* = 12)	
PAI-1 (ng/mL)	3.05 ± 0.56	5.23 ± 0.76	6.41 ± 1.36	0.0305*
Total adiponectin (mg/mL)	6.76 ± 0.78	7.48 ± 1.11	8.91 ± 2.3	NS
High molecular weight adiponectin (*μ*g/mL)	1.78 ± 0.26	2.33 ± 0.60	2.71 ± 1.54	NS
Leptin (ng/mL)	28.66 ± 7.93	46.87 ± 7.96	55.93 ± 11.39	NS
TA/L ratio	1.17 ± 0.76	0.24 ± 0.05	0.53 ± 0.32	0.0340*
HMWA/L ratio	0.49 ± 0.40	0.07 ± 0.02	0.26 ± 0.22	0.0442^#^

Data are presented as means ± SE. NS, not significant. *Established T2D versus controls/newly diagnosed T2D. ^#^Newly diagnosed/established T2D versus controls. IL-6: interleukin 6; TNF-*α*: tumor necrosis factor alpha; PAI: plasminogen activator inhibitor 1; TA/L: total adiponectin/leptin ratio; HMWA/L: high molecular weight adiponectin/leptin ratio.

**Table 4:** Measures of *β*-cell function and insulin sensitivity as per HOMA2 IR.

Measurement	Controls	Newly diagnosed T2D	Established T2D	*P* value
	(*n* = 15)	(*n* = 15)	(*n* = 15)	
HOMA2 %B	105.16 ± 10.63	118.02 ± 13.30	68.31 ± 11.0	0.0126^$^
HOMA2 %S	84.93 ± 8.19	60.03 ± 10.22	135.09 ± 37.85	NS
HOMA2 IR	1.41 ± 0.20	2.55 ± 0.52	2.67 ± 0.88	NS
HOMA2 %B C-peptide	111.42 ± 6.85	120.74 ± 10.31	85.15 ± 12.57	0.0473^$^
HOMA2 %S C-peptide	77.13 ± 9.81	135.79 ± 87.13	68.09 ± 17.06	NS
HOMA2 IR C-peptide	1.57 ± 0.17	2.71 ± 0.38	2.92 ± 0.75	NS

Data are presented as means ± SE. ^$^Newly diagnosed T2D versus controls/established T2D.

**Table 5:** Significant correlations between adipokines and measures of autonomic function.

Variable	Variable	Spearman	*P* value
IL-6	sdNN baseline	−0.3619	0.0217

TA/L ratio	TSP baseline	0.3519	0.0191
	sdNN baseline	0.2943	0.0525
	rmSSD baseline	0.2958	0.0512

HMWA/L ratio	LFA/RFA ratio	−0.4185	0.0042
	TSP baseline	0.3934	0.0082
	rmSSD baseline	0.3218	0.0332

HMW adiponectin	LFA/RFA	−0.5192	0.0003
